# Evaluation of Sleep Disturbances in Patients with Nocturnal Epileptic Seizures in a Romanian Cross-Sectional Study

**DOI:** 10.3390/healthcare10030588

**Published:** 2022-03-21

**Authors:** Réka Szabó, Florica Voiță-Mekereș, Cristina Tudoran, Ahmed Abu-Awwad, Mariana Tudoran, Petru Mihancea, Codrin Dan Nicolae Ilea

**Affiliations:** 1 Department of Neurological Rehabilitation, Municipal Clinical Hospital, 410469 Oradea, Romania; reka.szabo.85@gmail.com; 2 Doctoral School, Faculty of Medicine and Pharmacy, University of Oradea, 1 December Square, 410068 Oradea, Romania; petru.mihancea@yahoo.com (P.M.); codrin.ilea@csud.uoradea.ro (C.D.N.I.); 3 Department of Morphology, Faculty of Medicine and Pharmacy, University of Oradea, 1 December Square, 410068 Oradea, Romania; 4 Department VII, Internal Medicine II, University of Medicine and Pharmacy “Victor Babes” Timisoara, E. Murgu Square, Nr. 2, 300041 Timisoara, Romania; tudoran.mariana@umft.ro; 5 Center of Molecular Research in Nephrology and Vascular Disease, Faculty of Medicine, University of Medicine and Pharmacy “Victor Babes” Timisoara, E. Murgu Square, Nr. 2, 300041 Timisoara, Romania; 6 County Emergency Hospital, L. Rebreanu Str., Nr. 156, 300723 Timisoara, Romania; 7 Department XV—Orthopedics Traumatology, Urology, and Medical Imaging Internal Medicine II, Faculty of Medicine, University of Medicine and Pharmacy “Victor Babes” Timisoara, E. Murgu Square, Nr. 2, 300041 Timisoara, Romania; ahm.abuawwad@umft.ro

**Keywords:** sleep disturbances, nocturnal epileptic seizures, electroencephalography

## Abstract

(1) Background: Based on the premise that epilepsy is frequently associated with hypnopathies, in this study we aim to analyze the prevalence of sleep disturbances among patients with epilepsy, with exclusively or predominantly nocturnal seizures, in relation to demographic factors as well as clinical and electroencephalography (EEG) aspects. (2) Methods: 69 patients with nocturnal epilepsy were included in our study. Sleep disturbances were measured with the Pittsburgh Sleep Quality Index (PSQI) questionnaire, followed by a long-term video-EEG monitoring during sleep. We analyzed the PSQI results in relation to patients’ gender and age and determined the correlations between the PSQI scores and the modifications on video-EEG recordings, in comparison to a control group of 25 patients with epilepsy but without nocturnal seizures. (3) Results: We found a statistically significant difference between the PSQI of patients with nocturnal seizures compared to those without nocturnal epileptic manifestations. In the experimental group, the mean PSQI score was 7.36 ± 3.91 versus 5.04 ± 2.56 in controls. In women, the average PSQI score was 8.26, whilst in men it only reached 6.41, highlighting a statistically significant difference between genders (*p* ˂ 0.01). By examining the relationships between the PSQI scores and certain sleep-related factors, evidenced on the nocturnal video-EEG, we found a statistically significant difference between PSQI values of patients who reached the N2 stage, and those who reached the N3 stage of nonrapid eye movement (NREM) sleep, highlighting that those with a more superficial nocturnal sleep also had higher PSQI scores. There were no statistically significant differences regarding the PSQI scores between patients with or without interictal epileptiform discharges, and also in the few patients with nocturnal seizures where we captured ictal activity. (4) Conclusions: we evidenced in this study a poor quality of sleep in patients with nocturnal epilepsy, mostly in women, independent of age. We observed that sleep disturbances were due to superficial and fragmented sleep with frequent microarousals, not necessarily caused by the electrical epileptiform activity.

## 1. Introduction

Sleep disturbances are common in people with epilepsy, especially in those with nocturnal seizures [1].

The bidirectional interrelationship between sleep disorders and epilepsy has been frequently described in the medical literature [2,3] The frequency of seizures, their severity, and side effects of antiepileptic drugs may change sleep patterns and decrease the quality of sleep [4]. The occurrence of seizures can exert profound effects on the sleep architecture, lasting much longer than the postictal period [5], sleep disruption representing one of the major risk factors for the likelihood of recurrent seizures [6].

On the other hand, it is known that sleep is a potent activator of the interictal epileptiform discharges. There are two main sleep stages, nonrapid eye movement (NREM) sleep, and rapid eye movement (REM) sleep. Typically, epileptiform discharges are likely to propagate during NREM sleep, as this is when we see a more synchronized sleep architecture. In contrast, during REM sleep, there are asynchronous cellular discharge patterns that make epileptic EEG potentials less likely to propagate. NREM sleep is divided into stages 1, 2, and 3, representing a continuum of relative depth [7,8].

The mechanism of these interactions is strongly related to circadian rhythm regulation. The underlying molecular mechanisms are cell-autonomous transcription–translation feedback loops, comprising a set of core-clock genes. These genes not only regulate the circadian rhythms, but also contribute to the epileptic susceptibility. The core-clock genes may disrupt the neuronal inhibitory and excitatory balance rhythmically, leading to seizure periodicity [9,10].

The interrelation between epilepsy and sleep is determined by multiple factors, but they are insufficiently analyzed in the routine assessment of patients during the evaluation process. By using a subjective questionnaire-based method combined with an objective tool such as a long-term video-electroencephalography (EEG) recording, and analyzing the information offered by these two types of evaluations with their versatile correlations, we could contribute to superior monitoring of patients with epilepsy and with sleep disturbances.

The objective of this study is to analyze the prevalence of sleep disturbances among patients with epilepsy who have exclusively or predominantly nocturnal seizures, as well as the quality of sleep of these individuals, in relation to demographic factors and clinical and electroencephalography (EEG) aspects.

## 2. Materials and Methods

Study population: From a total of 142 patients with epilepsy who attended our Epilepsy Center in Oradea, Romania, between December 2016 and July 2020, we selected 69 adult patients with a known diagnosis of nocturnal epilepsy, and a control group of 25 subjects diagnosed with epilepsy but without nocturnal seizures, randomly selected during this timeframe. Given our sample size and group distribution, at alpha = 0.05, this control group offered a power of 89.52% for our study. They were all of Caucasian ethnicity. A signed informed consent form was obtained from all of the participants.

Inclusion criteria: adult patients over 18 years old, but under 85 years of age, with a confirmed diagnosis of nocturnal or mixed-type epilepsy (predominantly nocturnal but also minimum one or more diurnal seizures), who were willing and able to provide written informed consent.

Exclusion criteria: patients with a history of chronic respiratory disease, including obstructive sleep apnea, current smokers, subjects with progressive neurological disorders and those having psychiatric diseases or persistent insomnia, and individuals with shift-work history, were excluded from the study, because they could bias the results.

### Methods

Data were collected during the same period of time based on questionnaires administered during a single interview, including patients’ demographic characteristics, and a sleep questionnaire as well. Initially, each patient was evaluated by their attending physician, a board-certified neurologist, and, afterward, they all were evaluated by a second board-certified neurologist from our clinic before being considered eligible to be included in this study.

The validated Romanian version of the PSQI (Pittsburgh Sleep Quality Index) questionnaire was used for information collection. The PSQI is a tool employed to assess the quality and patterns of sleep during the last month by determining several factors: the subjective sleep quality, the sleep latency, the sleep duration, the habitual sleep efficiency, the existence of sleep disturbances, the use of hypnotic medication, and daytime dysfunction. The scoring of answers was based on the 4-point Likert scale, ranging from 0 to 3, whereby 3 reflects the negative extreme on the scale. A global score of 5 or greater indicates poor sleep [11].

Afterward, a nocturnal video-EEG, of a minimum 8 h duration was performed.

We used video-EEG monitoring which is considered an alternative for polysomnography to study the sleep architecture. We wanted to evaluate the epileptiform activity, which is more accurate in the 10–20 system registration on long-term video-EEG than in polysomnography, where we employ less EEG electrodes (typically 8). The polysomnography by employing many channels connected to the body may induce more discomfort to the patients and the subjects’ movements could interfere with the EEG channels and bias the results [12,13].

Technical aspects of the procedure: 21 electrodes were placed on the patients’ scalp to detect the electrical activity of the brain. The results were then transmitted to a machine, which represented them graphically on the monitor of a computer. Different parts of the brain were assessed, since these electrodes were positioned across the scalp in a mathematically precise manner. We utilized the 10–20 system, which is an internationally recognized and standardized method, to describe and apply the location of scalp electrodes during the EEG examination. A camera was employed to visually record the patients’ behavior, while, at the same time, the EEG was continuously recording their brain activity. During this process, patients’ vital signs and the appearance of clinical or electrical seizure activity were closely monitored and recorded on a computer These recordings offered us a more accurate possibility to compare the patients’ physical behavior with all types of electrical activities recorded on the EEG. The EEG was performed during awake, drowsy, and/or sleepy states. Provocative measures such as hyperventilation and photic stimulation were used to induce epileptiform activity.

Interpretation of video-EEG results: This was performed by the same neurologist with competency in EEG interpretation, the purpose being to avoid interexaminer reliability. The baseline electrical activity of the brain in an awake state, then the sleep architecture and the pathological epileptiform activity (ictal or interictal) were analyzed in detail, in correlation with the clinical manifestations.

Sleep was scored following the criterias from the AASM Manual for Sleep Scoring and Associated Eventes, according to the different stages: Stage W (wakefulness), Stage N1 (NREM 1), Stage N2 (NREM 2), Stage N3 (NREM 3), and Stage R (REM). Sleep was scored using 30 s epochs, starting with the first epoch acquired. Each epoch was scored based on the greatest sleep stage amount comprised within that time period. If three or more stages were present, the first step was to determine if there was more wake or sleep. If more sleep was present, then the epoch was assigned according to the sleep stage that occurred during the majority of that epoch. We have scored an epoch as stage W when more than 50% of that epoch had alpha rhythm over the occipital region. We have scored stage N1 when alpha rhythm was attenuated and replaced by low-amplitude, mixed-frequency activity for more than 50% of the epoch and we have noticed the presence of vertex sharp waves. Stage N2 was scored when we have noticed one or more K complexes or trains of sleep spindles occuring for at least half of the studied epoch. Stage N3 was scored when minimum 20% of an epoch consisted of slow wave activity. Stage R was scored when the following criteria were present: low amplitude, mixed-frequency EEG, low chin EMG tone and rapid eye movements. Arousals were scored during any stage of sleep when an abrupt shift of EEG frequency (alpha, theta or cycles ≥ 16 Hz, excepting spindles) that lasted at least 3 s was preceded by a minimum of 10 s stable sleep occured [14,15]. Finally, each patient received a detailed report about the events and further recommendations based on the conclusions of this evaluation.

Data analyses: For the statistical analysis of our data, we employed the R software v.4.1.2. R is available as free software under the terms of the Free Software Foundation’s GNU General Public License in source code form. R is a language and environment for statistical computing and graphics which provides a wide variety of techniques and is highly extensible. Continuous variables were presented as a mean with standard deviation (SD). We used the Shapiro–Wilk normality test, which is one of three general tests designed to detect all deviations from normality. The test rejects the hypothesis of normality when the *p*-value is less than or equal to 0.05. Considering that the results of the Shapiro–Wilk test showed a non-Gaussian distribution, we used further nonparametric tests for our analysis. The correlation between parameters was tested by Spearman’s rank-order correlation. Pearson’s chi-squared test was used to determine whether there is a statistically significant difference between the expected and observed frequencies in one or more categories of a contingency table. Fisher’s combined probability test was used to combine the results from several independent tests bearing upon the same overall hypothesis. *p* values under 0.05 were considered to indicate statistically significant differences. To assess the impact of several confounding factors on the variance of continuous variables we built a multivariate regression model by using the “backward elimination” method, and the model was validated based on the accuracy of prediction and R squared. In the final regression equations, the predictors were accepted according to a repeated backward-stepwise algorithm (inclusion criteria *p* < 0.05, exclusion criteria *p* > 0.10) to obtain the most appropriate theoretical model to fit the collected data.

The Local Scientific Research Ethics Committee of the Epilepsy Center in Oradea, Romania approved the design and methodology of our study (No. 21031/28 July 2020).

## 3. Results

Throughout the study period, based on evaluation and video-EEG performing, a total of 69 adult patients with epilepsy were eligible for participation in our research. All of them were already diagnosed with nocturnal or mixed-type epilepsy and came for monitoring or further evaluation of their neurological status. In total, 35 of them were women (50.72%) and 34 men (49.27%), the gender distribution slightly favoring women. Referring to the distribution by age, the youngest patient was 18 years old, and the oldest was 83 years old. The average age was 46.45 ± 18.65 years in women and 45.53 ± 17.1 years in men, with no statistically significant difference between the median age of women and men (*p* = 0.83). All data were compared with a control group comprising 25 subjects diagnosed with epilepsy but without nocturnal seizures, Table 1. These patients were aged between 20 and 80 years, the mean age being 52.33 ± 16.15 years in women and 57.23 ± 16.58 in men.

Referring to the distribution by their geographical area of origin in the experimental group, 44 patients (63.76%) were from an urban setting and 25 (36.23%) from rural areas. In the control group we had 17 subjects (68%) from an urban background and 8 (32%) from rural areas, Table 1.

Referring to patient history we had followed the time since the diagnosis of epilepsy was made, the characteristics of the seizures and the response to medication. In the experimental group, 8 patients were diagnosed 20 or more years ago, 13 subjects were diagnosed in the interval of the last 10–19 years, 21 patients in the interval of the last 5–9 years, and the remaining 27 subjects were diagnosed in the last 4 years. In the control group, 2 patients were diagnosed 20 or more years ago, 4 subjects were diagnosed in the interval of the last 10–19 years, 8 patients in the interval of the last 5–9 years, the remaining 11 subjects were diagnosed in the last 4 years. We have studied the seizure frequency per year and we have found that in the experimental group 15 patients had one seizure per year, 14 subjects had two seizures annually, 30 patients had more than three seizures per year and 10 subjects were seizure-free in the last 5 years. In the control group, 4 patients had one seizure per year, 6 subjects had two seizures annually, 13 patients had more than three seizures per year and 2 subjects were seizure-free in the last 5 years. Referring to seizure classification, about 47.82% of the patients in the experimental group had focal epilepsy, 37.68% generalized epilepsy, and 14.49% of them had epilepsy of an unknown type. We also analyzed the seizure types in the control group: 60% of the patients had focal epilepsy, 24% generalized epilepsy, and 16% of them had epilepsy of an unknown type. With regards to the use of medication, 60.86% of the patients in the experimental group received monotherapy and 39.13% received polytherapy (two or more drugs). In the control group, 60% used monotherapy and 40% polytherapy. In the experimental group, 34.78% of the patients had drug-resistant epilepsy versus 33% in the control group; see Table 2.

Afterward, we analyzed the patients’ bedtime routine, specifically, when they tend to go to sleep each night, and observed that the hour distribution was as follows: most patients, precisely 28 (40.58%), went to bed at 10 p.m., followed by 25 subjects (36.23%) who went to bed at 11 p.m. Of the remaining subjects, 8 (11.59%) had chosen to go to bed at midnight, and only 5 (7.25%) went to bed at 9 p.m. The other patients, 1 in each category, had bedtime hours at 8 p.m., 1 a.m. and 2 a.m, respectively.

We analyzed the distribution of bedtime hours by gender and we found that 18 women in the experimental group went to bed at 11 p.m., followed by 12 women who had bedtime hours at 10 p.m., only 3 went to bed at 9 p.m., and 2 women at midnight, none of them after midnight. The majority of men, namely 26, went to bed at 10 p.m., 7 had bedtime hours at 11 p.m. and 6 at midnight, and only 2 men at 9 p.m., The other men, one in each category, went to bed at 8 p.m., 1 a.m. or 2 a.m. In the control group nobody went to sleep before 8 p.m. or after midnight. One woman went to bed at 9 p.m., four at 10 p.m., five at 11 p.m. and the remaining two women at midnight. Two men choose to go to sleep at 9 p.m., five at 10 p.m., three at 11 p.m. and three at midnight.

We compared the bedtime hours of patients with nocturnal seizures to those with epilepsy but without nocturnal seizures, and found that most of patients from each group went to bed at 10 p.m. (28 patients in the experimental group and 9 in the control group), followed by patients in both groups who went to bed at 11 p.m. (25 subjects in the experimental group and 8 in the control group), then patients who had chosen to go to bed at midnight (8 versus 5). Three patients in each group had their bedtime hour at 9 p.m., one patient in the nocturnal seizure group went to bed at 8 p.m., one from the same group at 1 a.m. and another one at 2 a.m.; from the control group, none of the patients went to bed before 8 p.m. or after midnight, see Table 3.

The bedtime hour is not the same as the time of falling asleep, because many patients spent quite a long time until beginning their night’s sleep. We have studied how many hours they slept in total because we knew that this could influence not only their sleep quality, but also their daily performance: to stay awake while performing biological needs or engaging in social activities and to avoid daytime sleepiness, which was frequent in the study group based on the PSQI subpoint.

The average sleep duration, based on the PSQI questionnaire, was 7.35 ± 1.31 in the experimental group and 7.12 ± 0.88 in controls. We did not find a statistically significant difference between the two groups (*p* = 0.34).

Afterward, we analyzed the results of the PSQI questionnaire score, depending on patients’ demographic characteristics, in the experimental group versus the control group.

We found a statistically significant difference between the PSQI of patients with nocturnal seizures compared to those without nocturnal epileptic manifestations. The PSQI was higher with a mean value of 7.36 ± 3.91 in those of nocturnal seizures; however, the control group’s PSQI value of 5.04 ± 2.56 demonstrates that epileptic patients without nocturnal seizures also had sleep disturbances.

The PSQI average value in women with nocturnal seizures was of 8.26 ± 4.46, whilst in men this only reached 6.41 ± 3.03, highlighting a statistically significant difference between genders in the experimental group. The *t*-test showed a *p*-value < 0.05. The mean PSQI score by geographical area of origin was 8.52 for rural residence and 6.70 for urban background (*p* = 0.06) failing to evidence statistically significant differences concerning this criterion. In the control group, we found similar results regarding gender distribution, which favored women—see Table 4.

Subsequently, after patients completed the PSQI questionnaire, we performed an overnight video-EEG registration. This detected significant changes in the sleep architecture of most patients with nocturnal epilepsy, including sleep fragmentation and frequent microarousals.

**Figure 1:**
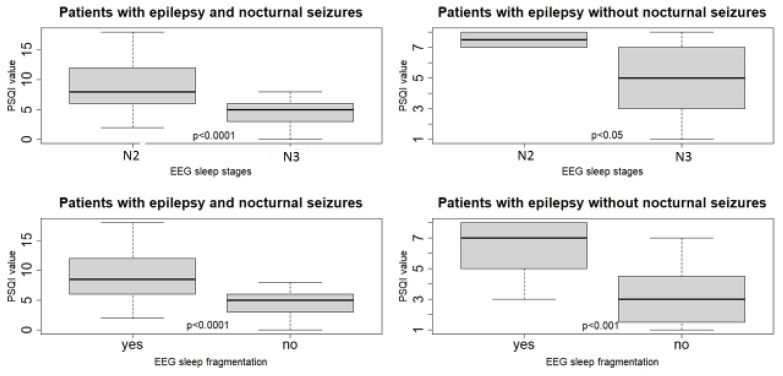
PSQI score distribution according to the deepest sleep stage reached. PSQI score distribution depending on sleep fragmentation by microarousals; comparison between the experimental group versus the control group.

The mean PSQI value was 9.17 in patients with fragmented sleep with microarousals, and of only 4.56 in patients without sleep fragmentation. The result of the *t*-test was *p* < 0.0001 (see Figure 1), evidencing a highly statistically significant difference between patients with fragmented sleep and those who had a continuous sleep concerning the average PSQI score. We found that 46 patients reached the N2 stage of non-REM sleep, and 26 patients slept deeper, reaching even the N3 stage of non-REM sleep. Mean PSQI value, depending on EEG sleep stages, was 9.05 for the N2 sleep stage and 4.58 for the N3 sleep stage. There was a highly statistically significant difference between patients who reached the N2 and the N3 stage of the non-REM sleep, concerning the average value of the PSQI score. The result of the *t*-test was *p* < 0.0001, see Figure 1. In the control group, the majority of patients reached a deeper sleep stage, N3. We observed that sleep fragmentation was also present in the control group, Figure 1.

Afterward, we analyzed the mean PSQI scores, depending on the interictal epileptiform activity of the selected patients. We concluded that those who had these discharges had a mean PSQI value of 7.65, while the group without discharges reached a value of 4.86. The *t*-test indicated a *p*-value 0.12; thus, there were no statistically significant differences between patients with or without interictal epileptiform discharges, as shown in Table 5.

We also analyzed the mean PSQI value, depending on ictal epileptiform discharges. The PSQI score was 7.57 for those with electrical seizures during the EEG monitoring, and a similar value of 7.31 was found in those without this type of ictal EEG finding (*p* = 0.81). In controls we did not evidence ictal activity, Table 5.

There was a statistically significant difference between the experimental and the control group referring to the deepest sleep stage reached. In patients with epilepsy and nocturnal seizures, only 37 (68%) achieved N3 sleep stage, compared to controls where 92% reached N3. The sleep fragmentation did not reveal a statistically significant difference between the two groups, and both had microarousals. The interictal epileptiform discharges were present in both groups with a statistically significant difference, Table 5.

We found that the PSQI score was higher in patients with a more superficial sleep who only reached the N2 sleep stage and had fragmented sleep with frequent microarousals. This finding was highly statistically significant in the experimental group but less so in the control group. Those who had epileptiform discharges had a higher PSQI score, but this was not statistically significant in the study populations. There was no major difference in the PSQI score of the epileptic patients with nocturnal seizures where we captured ictal activity, Table 6.

**Figure 2:**
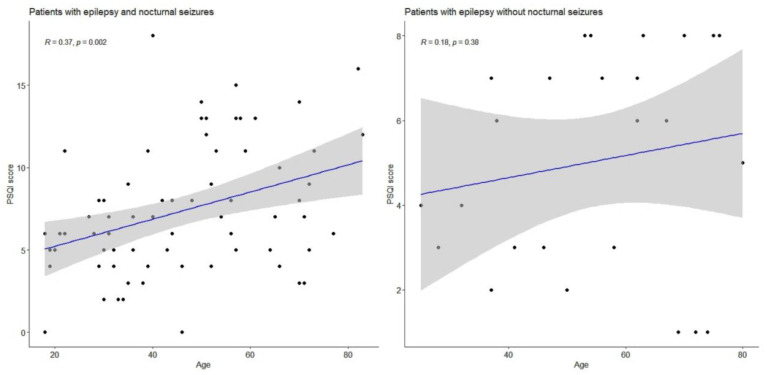
Correlation between PSQI score in relation to patients’ age in the experimental group versus control group; Spearman’s correlation.

We analyzed the correlation between the PSQI score and subjects’ age. We expressed the dispersion of the values on the histogram, which graphically illustrates the weak correlation between the PSQI result and patients’ age, Figure 2.

The PSQI and age variables are not part of normally distributed populations, according to Shapiro–Wilk test. We calculated a *p*-value of 0.0245 for the PSQI score and a *p*-value of 0.0302 for patients’ age. If the *p*-value was >0.05, then the variables would have a normal distribution according to the Shapiro–Wilk normality test.

Then, we tested the correlation between PSQI value and patients’ age using Spearman’s method. We obtained the following values: correlation (R) = 0.37 and *p* = 0.002, meaning a weak correlation between these variables. In the control group, we noticed an even more accentuated dispersion of values on the chart. The value of R = 0.18 represents a weak correlation between PSQI result and the age of the patient (*p* = 0.38); thus, this was not statistically significant.

To assess the impact of several confounding factors on the variance of continuous variables, we built a multivariate regression model by using the “backward elimination” method, initially introducing all predictor variables and then eliminating them one-by-one according to the *p* value. The final model showed that the occurrence of nocturnal seizures was influenced by the patient’s age, and PSQI score (as expected). The rest of the variables had no statistically significant influence on the occurrence of nocturnal seizures. Thus, we have a higher risk of nocturnal seizures with a factor of 1.31 in patients with a higher PSQI score by one unit if the age remains constant, as seen in Table 7.

## 4. Discussion

Our study evidenced that patients with nocturnal epilepsy have a worse sleep quality, likely due to superficial sleep and its fragmentation, with frequent microarousals, not necessarily caused by the electrical epileptiform activity [16,17]. The results obtained from the PSQI questionnaire, correlated with long-term video EEG monitoring, were able to confirm this aspect [18,19]. The scores for the subjective sleep quality and the sleep disruption questionnaires were significantly higher in our experimental patient group compared to the control [20,21].

Several studies from the medical literature support our findings, but in others, there are some contradictory results, which are outlined in Table 8. The study of Gutter et al. (2019) confirmed a higher prevalence of sleep disturbances in subjects with epilepsy than in controls. This resulted in lower quality of life (QoL) scores in both groups—people with epilepsy and controls—but more so in people suffering from epilepsy [22]. The study of Katami et al. concluded that sleep–wake habits and the frequency of most sleep disorders are similar in nonselected patients with epilepsy compared to controls [23]. Another study, by Krishnan et al., employed multiple sleep questionnaires on patients with epilepsy, accompanied by a routine EEG. They found that subjects with juvenile myoclonic epilepsy had significantly more sleep disturbances, characterized by excessive daytime sleepiness and disturbed nighttime sleep, despite adequate medications and reasonable seizure control [24].

A very recent study from 2021, published by Safarpour Lima et al., highlighted that sleep disturbances are common findings in patients with epilepsy, which may even become severe in some cases. Sleep disturbances were more common among patients with a higher seizure frequency and in patients with generalized seizures. They recommended a routine evaluation of sleep disorders among patients with epilepsy to improve their sleep quality. This study lacks the EEG evaluation of the whole sample group [25].

Jin Bo et al. published in 2020 an article debating the role of the long-term video-EEG monitoring and its clinical applications, evidencing its importance for our knowledge over the interaction between circadian rhythms, sleep, and epilepsy [10].

We compared the results obtained in our study with data from two meta-analyses referring to the different sleep disturbances in patients with epilepsy. Lin Zhang et al. studied the prevalence of obstructive sleep apnea in subjects with epilepsy and concluded that it is higher than in the general population. Additionally, their results suggest that the treatment of sleep apnea resulted in a reduction of seizures [26]. Another recent meta-analysis by Bergman et al., published in 2021, evidenced that patients with epilepsy had worse PSQI scores compared to controls. They mentioned that accurate data about the influence of age on sleep quality in epilepsy are still missing. An innovation in our study is that we described the correlations between the PSQI values and the age of patients with epilepsy. They recommend that in epilepsy, the sleep quality should be evaluated not only by questionnaires, but also by objective measures, which was done in our research with the nocturnal video-EEG [27].

We emphasized in our study the impact of the total PSQIscore, but the subscores of the PSQI showed different aspects of sleep quality. We found that age was an independent factor when analyzing PSQI total scores. Women with epilepsy and nocturnal seizures had higher PSQI total scores. The geographical place of origin had a limited influence on PSQI value. Our results illustrated the heterogeneity in the PSQI-defined poor sleep quality in individuals. In addition to the total scores, PSQI subscores should be analyzed in detail in further studies, mostly when significant sleep impairment is noticed. A possibility could be to subgroup changes in sleep duration, sleep difficulty, insomnia symptoms and the relationship between activity rhythm and depressive symptoms, respectively sleep medication use. Subgrouping the PSQI-defined poor-sleep-quality individuals could help to analyze the details of the type of sleep disturbances and thus to contribute to their management [28].

A limitation of our study is the relatively small sample size. Longitudinal studies, with more cases, as well as supplementary sleep tests are required, to solidify our findings, which should constitute a foundation for further detailed research. Another limitation of this paper is that we did not detail the potential side effects on sleep of the antiepileptic drugs taken by our patients; this could be part of future research.

We consider this modality of evaluation: firstly, a sleep questionnaire followed by a long-term video-EEG monitoring could represent a possibility for a more detailed evaluation of patients with nocturnal seizures regarding their sleep disturbances which have a major impact on their quality of life. We propose to complete the evaluation of patients with nocturnal epilepsy with sleep questionnaires and long-term video-EEG monitoring because these are widely available, noninvasive methods, and could reveal sleep disturbances leading to a poorer quality of life.

## 5. Conclusions

In sum, the results of our study evidenced a poor quality of sleep in patients with nocturnal epilepsy, mostly in women, independent of age. We noticed changes in sleep architecture, associated with a poor quality of sleep, in patients with nocturnal epilepsy. Our findings support the necessity of undertaking objective sleep tests for an early diagnosis of sleep disturbances in patients with epilepsy.

## Figures and Tables

**Table 1:** Demographic characteristics of patients.

Variables		Patients with Nocturnal Epilepsy N = 69 (100%)	Patients without Nocturnal Epilepsy N = 25 (100%)
Gender:	Women	35 (50.72%)	12 (48%)
	Men	34 (49.27%)	13 (52%)
Mean age (years)		46.00 ± 17.78	54.88 ± 16.23
Origin:	Urban	44 (63.76%)	17 (68%)
	Rural	25 (36.23%)	8 (32%)

**Table 2:** Clinical characteristics of patients.

Variables		Patients with Nocturnal Epilepsy N = 69 (100%)	Patients without Nocturnal Epilepsy N = 25 (100%)
Time since diagnosis (years)	≥20	8	2
	10–19	13	4
	5–9	21	8
	≤4	27	11
Seizure frequency (per year)	1	15	4
	2	14	6
	≥3	30	13
	seizure free *	10	2
Seizure classification	focal	33	15
	generalized	26	6
	unknown type	10	4
Antiepileptic medication use	monotherapy	41	15
	polytherapy **	28	10
Drug resistant epilepsy	yes	24	8
	no	45	17

Legend: * in the last 5 years; ** ≥2 drugs.

**Table 3:** Bedtime hour and sleep duration habits.

Variables		Patients with Nocturnal Epilepsy N = 69 (100%)		Patients without Nocturnal Epilepsy N = 25 (100%)	
		Women	Men	Women	Men
Bed time hour	8 p.m.	0	1	0	0
	9 p.m.	3	2	1	2
	10 p.m.	12	26	4	5
	11 p.m.	18	7	5	3
	12 p.m.	2	6	2	3
	1 a.m.	0	1	0	0
	2 a.m.	0	1	0	0
Sleep duration time (hours)	5	7		0	
	6	9		7	
	7	22		9	
	8	20		8	
	9	6		1	
	10	5		0	

Legend: p.m.—post meridiem; a.m.—ante meridiem.

**Table 4:** PSQI scores depending on gender and demographical data.

PSQI		Patients with Nocturnal Epilepsy N = 69 (100%)			Patients without Nocturnal Epilepsy N = 25 (100%)		
		Mean	SD	*p* Value for *t*-Test	Mean	SD	*p* Value for *t*-Test
Gender	Women	8.29	4.46	*p* < 0.05	6.58	1.88	*p* < 0.01
	Men	6.41	3.03		3.62	2.29	
Residence	Rural	8.52	3.84	*p* = 0.06	4.63	2.77	*p* = 0.61
	Urban	6.70	3.83		5.24	2.51	

Legend: PSQI—Pittsburgh Sleep Quality Index.

**Table 5:** Distribution of sleep stages and epileptiform discharges on video-EEG.

VideoEEG Findings		Patients with Epilepsy and Nocturnal Seizures N = 69 (100%)		Patients with Epilepsy without Nocturnal Seizures N = 25 (100%)		*p* Value
The deepest sleep stage reached	N2	43	62.32%	2	8%	Chi square test; *p* < 0.0001
	N3	26	37.68%	23	92%	
Fragmented sleep with microarousals	Yes	42	60.87%	14	56%	Chi square test; *p* = 0.85
	No	27	39.13%	11	44%	
Interictal epileptiform discharges	Yes	62	89.86%	18	72%	Fisher test; *p* < 0.05
	No	7	10.14%	7	28%	
Ictal epileptiform discharges	Yes	14	20.29%	0	0%	Fisher test; *p* < 0.05
	No	55	79.71%	25	100%	

**Table 6:** PSQI depending on the distribution of sleep stages and epileptiform discharges on video-EEG.

VideoEEG Findings		PSQI of Patients with Epilepsy and Nocturnal Seizures N = 69 (100%)			PSQI of Patients with Epilepsy, without Nocturnal Seizures N = 25 (100%)		
		mean	SD	*p* Value (*t*-Test)	mean	SD	*p* Value (*t*-Test)
The deepest sleep stage reached	N2	9.05	3.38	*p* < 0.0001	7.5	0.71	*p* < 0.05
	N3	4.58	2.14		4.83	2.55	
Fragmented sleep with microarousals	Yes	9.17	3.74	*p* < 0.0001	6.5	1.79	*p* < 0.001
	No	4.56	2.10		3.18	2.18	
Interictal epileptiform discharges	Yes	7.65	3.82	*p* = 0.12	5.78	2.26	*p* < 0.05
	No	4.86	4.01		3.14	2.41	
Ictal epileptiform discharges	Yes	7.57	3.55	*p* = 0.81			
	No	7.31	4.02		5.04	2.56	

Legend: PSQI—Pittsburgh Sleep Quality Index.

**Table 7:** Regression model evidencing the impact of age and PSQI score on the occurrence of nocturnal seizures.

	OR	2.5%	97.5%
(Intercept)	4.7870294	0.9762226	26.6911560
Age	0.9565057	0.9256186	0.9851304
PSQI score	1.3130424	1.1190367	1.5894642

**Table 8:** A comparison table of clinical studies which investigated the sleep disturbances in epilepsy.

No.	Author	Year	Study Design	Formulations Studied	Key Findings
1.	Gutter et al.	2019	Cross-sectional, case-control study about the prevalence of sleep disturbances in people with epilepsy and the impact of it on QoL	GSQS, MOSS, SDL and ESS to measure sleep and the SF-36 to measure QoL	Higher prevalence of sleep disturbances and lower QoL scores in people with epilepsy compared to controls
2.	Khatami et al.	2006	Prospective, case-control study about sleep–wake habits and disorders in epileptic subjects	ESS for excessive daytime sleepiness, SA-SDQ for sleep apnea and the UNS for narcolepsy	Sleep–wake habits and the frequency of most sleep disorders are similar in non-selected epilepsy patients as compared to controls
3.	Krishnan et al.	2012	Case control study to analyze the effect of epilepsy on sleep in patients with JME	EEG, neuroimaging and validated sleep questionnaires: ESS, PSQI and NIMHANS Sleep Disorders Questionnaire	Patients with JME have significant sleep disturbances, despite adequate medications and good seizure control
4.	Safarpour et al.	2021	Cross-sectional Persian study to assess the prevalence of common sleep disorders in patients with epilepsy	Validated Persian questionnaires were used to assess EEDS, RLS and insomnia	Sleep disturbance is a common finding in patients with epilepsy, which may become severe in some cases

Legend: QoL—quality of life; GSQS—Groningen Sleep Quality Scale; MOSS—Medical Outcomes Study-Sleep scale; SDL—Sleep Diagnosis List; SF-36—Short Form Health Survey; ESS—Epworth Sleepiness Scale; SA-SDQ—Sleep Apnea scale of the Sleep Disorders Questionnaire; UNS—Ullanlinna Narcolepsy Scale; JME—juvenile myoclonic epilepsy; EEG—electroencephalogram; NIMHANS—National Institute of Mental Health and Neurosciences; EEDS—excessive daytime sleepines; RLS—restless leg syndrome.

## Data Availability

Our data are available on Mendeley, at doi:10.17632/wpn9jvfmv7.1, 20 January 2022.
